# Comparative Analysis of 3D Bladder Tumor Spheroids Obtained by Forced Floating and Hanging Drop Methods for Drug Screening

**DOI:** 10.3389/fphys.2017.00605

**Published:** 2017-08-22

**Authors:** Robson L. F. Amaral, Mariza Miranda, Priscyla D. Marcato, Kamilla Swiech

**Affiliations:** Department of Pharmaceutical Sciences, School of Pharmaceutical Sciences of Ribeirão Preto, University of São Paulo São Paulo, Brazil

**Keywords:** 3D spheroids, RT4 bladder cancer cells, ultra-low attachment plates, hanging-drop plates, drug screening, cytotoxicity assays

## Abstract

**Introduction:** Cell-based assays using three-dimensional (3D) cell cultures may reflect the antitumor activity of compounds more accurately, since these models reproduce the tumor microenvironment better.

**Methods:** Here, we report a comparative analysis of cell behavior in the two most widely employed methods for 3D spheroid culture, forced floating (Ultra-low Attachment, ULA, plates), and hanging drop (HD) methods, using the RT4 human bladder cancer cell line as a model. The morphology parameters and growth/metabolism of the spheroids generated were first characterized, using four different cell-seeding concentrations (0.5, 1.25, 2.5, and 3.75 × 10^4^ cells/mL), and then, subjected to drug resistance evaluation.

**Results:** Both methods generated spheroids with a smooth surface and round shape in a spheroidization time of about 48 h, regardless of the cell-seeding concentration used. Reduced cell growth and metabolism was observed in 3D cultures compared to two-dimensional (2D) cultures. The optimal range of spheroid diameter (300–500 μm) was obtained using cultures initiated with 0.5 and 1.25 × 10^4^ cells/mL for the ULA method and 2.5 and 3.75 × 10^4^ cells/mL for the HD method. RT4 cells cultured under 3D conditions also exhibited a higher resistance to doxorubicin (IC_50_ of 1.00 and 0.83 μg/mL for the ULA and HD methods, respectively) compared to 2D cultures (IC_50_ ranging from 0.39 to 0.43).

**Conclusions:** Comparing the results, we concluded that the forced floating method using ULA plates was considered more suitable and straightforward to generate RT4 spheroids for drug screening/cytotoxicity assays. The results presented here also contribute to the improvement in the standardization of the 3D cultures required for widespread application.

## Introduction

Reproducing *in vitro* the complexity of a tumor for drug screening assays is considered a major challenge during drug development. Traditionally, *in vitro* cell-based assays are carried out using two-dimensional (2D) cell culture (Edmondson et al., 2014). However, most tumor cells in an organism, as wells as healthy cells in normal tissue, exist in a three-dimensional (3D) microenvironment. The 3D microenvironment is important since the phenotype and function of individual cells are strongly dependent on interactions with proteins of the extracellular matrix (ECM) and with neighboring cells (Abbott, 2003). Cells cultured under 2D conditions exhibit a significant reduction in cell-cell and cell-ECM interactions, limiting the ability of these cultures to mimic natural cellular responses *in vitro* (Lee et al., 2009). When cultured in 3D systems, cells are able to recover some characteristics that are critical for physiologically relevant cell-based assays. Since external stimuli dramatically affect the properties, behavior, and functions of cells, they may also affect the response of cells to the compounds being tested (Quail and Joyce, 2013; Smith and Kang, 2013; Yulyana et al., 2015).

Cells can be cultured in 3D employing scaffolds and/or scaffold-free techniques. The first method involves seeding the cells on an acellular matrix or dispersing them in a liquid matrix, which subsequently solidifies or polymerizes. These scaffolds are made of either biological-derived materials (Sutherland et al., 1971) or synthetic materials (Edmondson et al., 2014). Matrigel™, a mouse-derived reconstituted basement membrane (Souza et al., 2010), has been commonly used as biological-derived scaffolds for spheroids generation improving different tumor cell lines (Mouhieddine et al., 2015; Daoud et al., 2016). However, once Matrigel™ is an animal-derived ECM, it can potentially affect experimental results because it may contain endogenous growth factors that do not mimic human tumor environment (Stevenson et al., 2006). Alternatively, polymeric scaffolds using synthetic hydrogels such as poly(ethylene glycol) (PEG), poly(vinyl alcohol), and poly(2-hydroxy ethyl methacrylate) have been used to minimize the relatively poor reproducibility of biological-derived scaffolds (Fang and Eglen, 2017). On the other hand, scaffold-free systems do not require the use of any support to grow the cells, being the most widely used model (Benien and Swami, 2014; Jaganathan et al., 2014). Under appropriate conditions cells are induced to self-assemble into spheroids that are characterized by their round shape and ability to be maintained as free-floating cultures (Ivascu and Kubbies, 2006; Lin and Chang, 2008; Weiswald et al., 2015). One of the main advantages of this method is that multicellular spheroids can restore the cellular heterogeneity of solid tumors (Mueller-Klieser, 2000; De Sousa E Melo et al., 2013). This heterogeneity is a result of the lack of vascularization, which leads to poor diffusion of oxygen and nutrients, resulting in the formation of gradients (Thurber et al., 2008). Thus, proliferative cells are arranged toward the external zone of the spheroids, while the interior consists of a quiescent region resulting from the limited supply of oxygen, nutrients, and essential metabolites. In the inner region of the spheroid, the absence of oxygen leads to the development of a necrotic core with an acidic pH environment. This hypoxia results in indirect effects on tumor cells by affecting expression patterns (Francia et al., 2005; Shield et al., 2009; Hirschhaeuser et al., 2010). This is the same growth pattern as that observed in the initial phase of some solid tumors *in vivo* (Knuchel et al., 1988). Therefore, 3D spheroids models are able to re-establish the morphological, functional, and mass-transport properties of the corresponding tissue *in vivo* (Friedrich et al., 2009).

Scaffold-free spheroids can be generated by the forced floating method, the hanging drop method, or agitation-based approaches (Breslin and O'Driscoll, 2013). The forced floating method is carried out using uncoated plates (as polystyrene surface has low-adhesion properties) or plates that are coated with a hydrophilic polymer that suppresses cell-substrate interactions, such as ultra-low attachment plates. One advantage of this method is the possibility of automation that makes it suitable for high-throughput screening (Friedrich et al., 2009). In the hanging drop method, cells accumulate at the free liquid-air interface to form a single spheroid resulting from the inversion of the tray present in the plate. The gas exchange in spheroids produced by this method is relatively higher (Kelm et al., 2003) and possible effects on cells due to contact with substratum are avoided. However, spheroids need to be transferred to other standard plates for use in cell-based assays (Kelm et al., 2003).

Even though the use of 3D cultures has gained popularity, they have not yet replaced the classical 2D model in both industrial and academic research. There is still a demand for the demonstration of the robustness, reliability, and versatility of models (Hickman et al., 2014). The transition from the use of 2D cultures to 3D cultures will be effective when high levels of standardization are achieved (Hirschhaeuser et al., 2010). This depends on a thorough understanding of cellular behavior in 3D models. Here, we report a comparative analysis of 3D spheroids generated by forced floating (ultra-low attachment- ULA plates) and hanging drop (HD) methods. The RT4 human-bladder cancer cell line was used as a model. The morphological features of the spheroids, cell growth behavior, metabolism, and response to treatment with the anticancer drug doxorubicin were evaluated in both methods. Results were also compared to that seen under 2D culture conditions.

## Materials and methods

### Cell line and general culture conditions

The RT4 cell line derives from the explants of a transitional cell papilloma of the bladder (grade 1 carcinoma) and it was obtained from the Rio de Janeiro Cell Bank (BCRJ 0214). Cells were routinely cultured as a monolayer in T-75 flasks (Corning®, New York, USA) with McCoy's 5A Medium with L-glutamine (Sigma-Aldrich®, St. Louis, Missouri, USA) supplemented with 2.2 g/mL of sodium bicarbonate (Sigma-Aldrich®, St. Louis, Missouri, USA), 10% (v/v) Fetal Bovine Serum (Sigma-Aldrich®, St. Louis, Missouri, USA) and 1% (v/v) Penicillin-Streptomycin (10,000 U/mL) (Sigma-Aldrich®, St. Louis, Missouri, USA). The cells were kept in a humidified atmosphere of 5% CO_2_ in air at 37°C.

### Generation and characterization of 3D spheroid cultures

3D spheroids of RT4 cells were generated by two scaffold-free techniques: forced floating method using 96-well round-bottom Ultra Low Attachment (ULA) plates (Corning®, New York, USA) and hanging drop method using 96-well Perfecta3D® Hanging Drop (HD) Plates (3DBiomatrix, Ann Arbor, Michigan). Seeding concentrations of 0.5, 1.25, 2.5, and 3.75 × 10^4^ cells/mL were used to generate 3D spheroids in both methods. For ULA plates it used a working volume of 200 μL of culture medium per well and for HD plates a working volume of 40 μL/well per well. Fresh culture medium (10 μL/well) was added to the HD cultures every 2 days to compensate for losses caused by evaporation. For comparison purposes, RT4 cells were also cultured as a monolayer (2D) in treated 96-well flat-bottom plates (Corning®, New York, USA) at the same seeding concentrations testes in 3D cultures. The morphology of the spheroids, cell growth and metabolism were characterized over a 7-day culture period in quadruplicate.

#### Morphology

Images of all spheroids were taken daily for 7 days using a camera attached to a Ti-S Inverted Microscope (Nikon Instruments Inc., Holland) with a 5x objective. All images were analyzed using the open-source software ImageJ (National Institutes of Health, Bethesda, Maryland, USA), and a macro was written to automate the process (Macro S1) as described by Ivanov et al. (2014). Diameter and all shape parameters were classified according to the image analysis function of ImageJ. Feret's diameter was used in the estimation of the mean diameter of the spheroids. The parameter “roundness” is a measure of how close the shape of the 2D spheroid image approaches a circle (the shape's gross features). The “Solidity” function, which is an indicator of the roughness of the spheroidal surface, was determined in order to assess its regularity. The ImageJ parameter “Circularity” (Cir) was used to calculate the Sphericity Index (SI), which determines how close to a spherical geometry shape our samples are, according to the Equations (1) and (2) (Kelm et al., 2003).

(1) $$\begin{array}{r} \text{Cir}=\left(\frac{4\pi \times Area}{Perimeter^{2}}\right) \end{array}$$

(2) $$\begin{array}{r} \text{SI}=\sqrt{Cir}\; \end{array}$$

Curves of diameter or shape parameters were plotted against time using Origin graphing and data analysis software (OriginLab, Massachusetts, USA). Data were displayed as mean ± SEM (*n* = 4).

3D spheroids were also analyzed by scanning electron microscopy (SEM) on the 3rd day. Spheroids were selected from cultures initiated with 12.5 × 10^4^ cells/mL in the ULA plates, and from cultures initiated with 3.75 × 10^4^ cells/mL in the HD plates and then fixed in 2.0% (v/v) glutaraldehyde (Sigma-Aldrich®, St. Louis, Missouri, USA) for 1 h. After fixation, the samples were washed thrice with cacodylate buffer (0.2 M, pH 7.2; Sigma-Aldrich®, St. Louis, Missouri, USA) for 10 min, immersed in osmium tetroxide (1%; Sigma-Aldrich®, St. Louis, Missouri, USA) for 1 h, dehydrated using a graded series of ethanol (50, 70, 95%) for 15 min each, followed by twice in 100% ethanol, and dried by using the critical-point-dryer, CPD030 (BAL-TEC AG, Liechtenstein). The dried samples were sputter-coated with gold, and examined under the SEM, High Vacuum JSM-6610LV (JEOL, Japan).

#### Cell growth and viability

For characterization purposes, the number of cells present in each spheroid was estimated in quadruplicate using a Neubauer chamber (Boeco, Germany) under a LX 400 microscope (Labomed Inc., Los Angeles, California, USA). For this analysis, spheroids were first dissociated using a solution of 0.15% trypsin and 0.06% of Ethylenediamine tetraacetic acid (EDTA; Sigma-Aldrich®, St. Louis, Missouri, USA) at 37°C for 15–20 min. During characterization, cell viability was determined in quadruplicate by subtracting dead cells stained with 0.4% (v/v) Trypan Blue solution (Sigma-Aldrich®, St. Louis, Missouri, USA) from the total number of cells. Curves of cell concentration or viability were plotted against time using Origin graphing and data analysis software (OriginLab, Massachusetts, USA). Data were displayed as mean ± SEM (*n* = 4). The fold-increase numbers (FI) for each condition were determined by dividing the final concentration of cells by the initial one.

#### Apoptosis analysis

After spheroid dissociation, single-cell suspensions were treated with Annexin V Binding Buffer 1X (BD Biosciences, San Jose, California, USA), and stained with 5- Fluorescein Isothiocyanate (FITC) conjugated with Annexin V solution (BD Biosciences, San Jose, California, USA) and Propidium Iodide solution (PI) (BD Biosciences, San Jose, California, USA) in order to estimate the percentage of apoptotic cells by using the FACSCalibur flow cytometer (BD Biosciences, San Jose, California, USA). Samples were analyzed in quadruplicate.

#### Cell metabolism

During the 7-day culture, samples of the supernatant from the ULA, HD, and 2D cultures were collected and stored at −20°C. The concentrations of glucose, glutamine, and lactate in the supernatant were determined in triplicate using an automatic 2700 YSI Biochemistry Analyzer (Yellow Springs Instruments, Yellow Springs, Ohio, USA). Curves of glucose, glutamine or lactate concentration were plotted against time using Origin graphing and data analysis software (OriginLab, Massachusetts, USA). Data were displayed as mean ± SEM (*n* = 3).

### Drug sensitivity assay

In order to compare the anticancer effects in 3D spheroids and 2D monolayers, cultures were treated with six different concentrations of doxorubicin: 4.0, 2.0, 1.0, 0.5, 0.25, and 0.125 μg/mL in a final volume of 100 μL. Doxorubicin stock solution (1.0 mg/mL) was diluted in McCoy's 5A culture medium to a final concentration of 2X immediately before use. Cultures were also treated with 20% of Dimethyl sulfoxide (DMSO) as a positive control, and fresh culture medium without drug was used as the negative control. Seeding concentrations of 0.5 and 1.25 × 10^4^ cells/mL, and 2.5 and 3.75 × 10^4^ cells/mL were used for ULA plates and HD plates respectively. After 72 h, 3D spheroid cultures were treated with doxorubicin solution by replacing 50% of the supernatant with drug-supplemented culture medium following the protocol established by Friedrich et al. (2009). After treatment, cultures were incubated for 24 h, and analyzed by ATP-based cell viability assays using CellTiter-Glo® 3D (Promega, Madison, Wisconsin, USA). A volume of CellTiter-Glo® 3D solution equal to the volume of the cell culture medium in each well was added. To induce cell lysis, the 96-well plates were vigorously shaken for 5 min, and incubated at room temperature for an additional 25 min. Then, the supernatant was transferred to an opaque-walled multiwell plate, and the luminescence was recorded using a Synergy™ 2 Multi-Mode Microplate Reader (BioTek®, Winooski, Vermont, USA) an integration time of 0.25–1.0 s per well. The same protocol was followed for 24-h monolayer (2D) cultures seeded with the same cell concentration tested in 3D cultures. Six samples per condition (*n* = 6) were considered for each analysis.

#### IC_50_ estimation

Cell viability (Y) was calculated using the luminescence values of the samples (S), and of the positive (PC) and negative controls (NC) according to the Equation (3):

(3) $$\text{Y}=[(\frac{S-PC}{NC-PC})\;\times \;100)]$$

The inhibitory dose-response curves were plotted by taking the logarithms of the concentrations of the drug tested along X-axis and cell viability along Y-axis. Using Prism 7 software (GraphPad Software, Inc., La Jolla, California, USA) the parameter “Log (inhibitor) vs. normalized response” was chosen in order to estimate the IC_50_ value (concentration of a drug that gives half-maximal inhibitory response).

### Statistical analysis

All statistical analyses in this work were performed using Prism software version 7.0 (GraphPad Software, Inc., La Jolla, California, USA). The fold-increase number (FI) and the percentage of apoptotic cells in 2D and 3D cultures were compared using One-way ANOVA statistic method with *post-hoc* test Tukey HSD and with *P* < 0.05 considered significant (*n* = 4). The IC_50_ values obtained in 2D and 3D cultures were analyzed using Extra-sum-of-squares statistic method with *F*-test and with *P* < 0.05 considered significant. To assess the reliability of this statistical analysis, the confidence interval was also determined.

## Results and discussion

Several studies have shown that the cell-cell, cell-ECM, and cell-microenvironment interactions in 3D spatial arrangement can affect many cellular functions including cell proliferation, organization, and responses to external stimuli leading to the development of several features that are unique to tumor spheroids (Zanoni et al., 2016). These features are supposed to be different not only between 3D and 2D conditions, but also among 3D cell cultures generated by different methods. For this reason, the morphology parameters and growth/metabolism of spheroids generated by the two different methods, forced floating using ULA plates (ULA) and hanging-drop (HD), were first characterized, and then subjected to evaluation of cytotoxicity.

### RT4 cells form regular and round-shaped spheroids within 24–48 h of 3D culture

**Figure 1:**
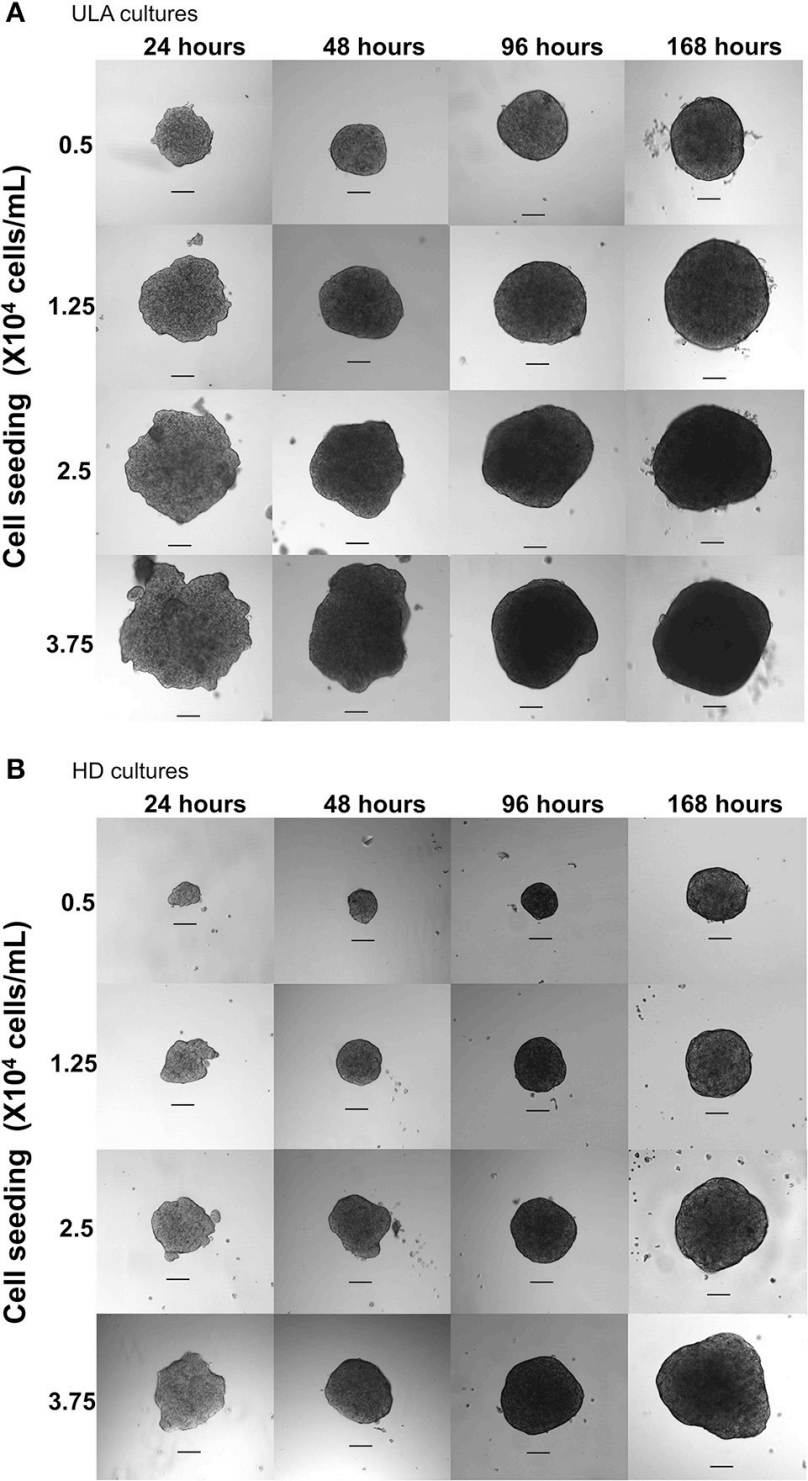
Phase contrast microscopic images of 7-day RT4 spheroid culture in ULA **(A)** and HD plates **(B)** with different cell-seeding concentrations (4X objective, scale bar = 100 μm).

Images obtained by using the phase contrast microscope showed the spontaneous formation of a single cell aggregate per well both in ULA and HD plates in the first 24 h of culture in all cell-seeding concentrations evaluated (Figure 1). After 48 h, a decrease was observed in the size of the cell aggregates, which became more compact, forming solid spheroids. Zanoni et al. (2016) named the time interval for this process as “spheroidization time” that varies depending on the cell type and culture conditions. The RT4 spheroids generated using the ULA and HD plates presented a round-type morphology. According to Kenny et al. (2007), this morphology is related to strong cell-cell adhesion.

**Figure 2:**
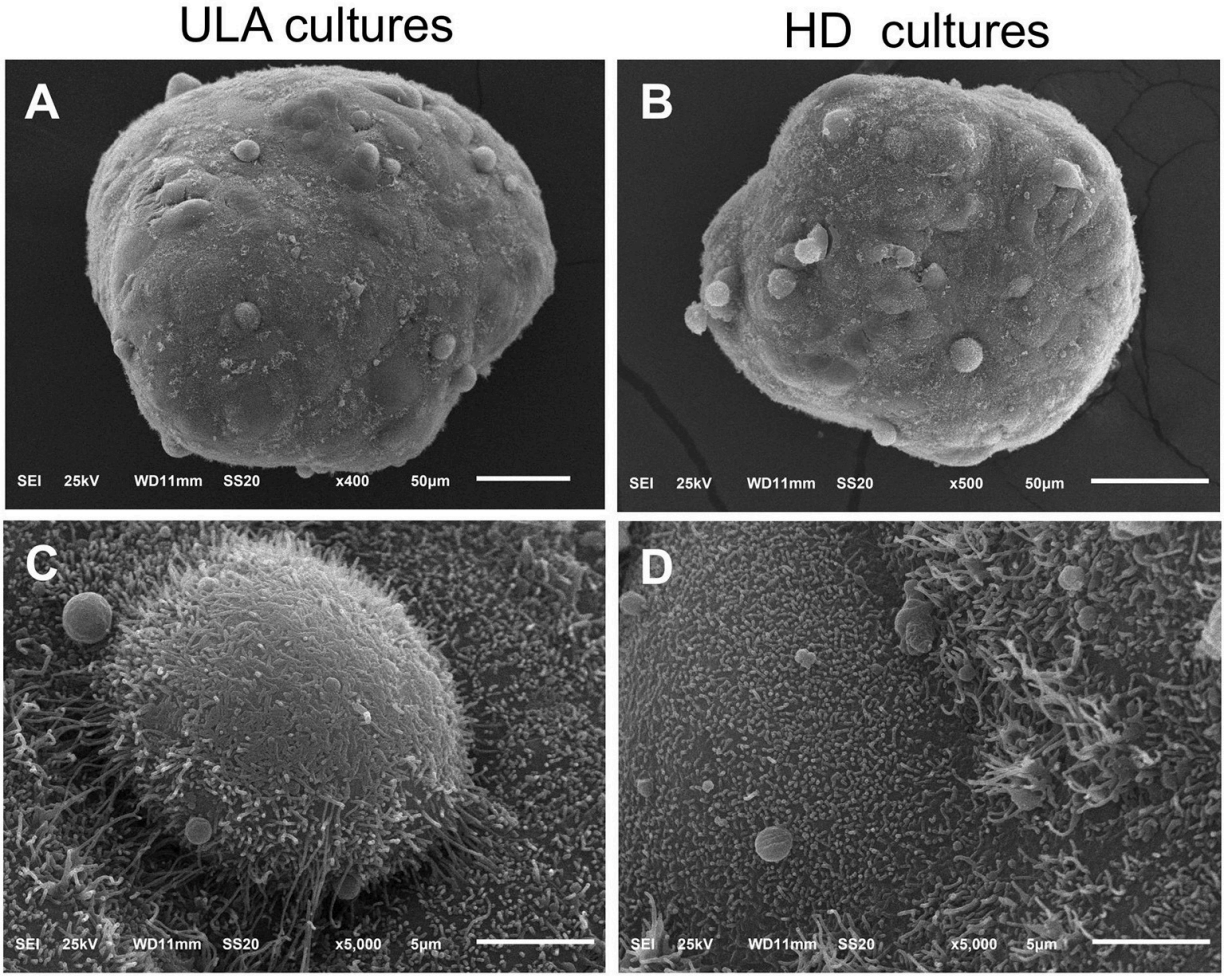
Scanning electronic microscopic images of 72-h RT4 spheroids from ULA **(A,C)** and HD plates **(B,D)**. **(A,B)**: 400/500X objective, scale bar = 50 μm; **(C,D)**: 5000X objective, scale bar = 5 μm.

SEM images of spheroids from both ULA and HD plates showed that the surface of the spheroids was smooth and continuous (Figures 2A,B). The smooth nature of the surface is attributed to high ECM secretion that conceals individual cells in most parts of the spheroidal surface (Lin et al., 2006). The surface of spheroids from both the ULA and HD plates were covered with plasma membrane projections called microvilli (Figures 2C,D). Surfaces similar to those of tissues with apical microvilli are typical of transitional epithelial cells present in the bladder (Kaufman et al., 2009). Moreover, these microvilli project outward to a high degree in a type of transitional cell carcinoma known as papillary carcinoma, from which the RT4 cell line is derived (Jacobs et al., 1976). Microvilli are not often present in cells cultured conventionally in monolayers (Gardner and Herbst-Kralovetz, 2016; McConkey et al., 2016; Wang et al., 2016), suggesting that RT4 spheroids can reproduce the characteristic features of a papillary tumor *in vitro* better than monolayer cell cultures.

**Figure 3:**
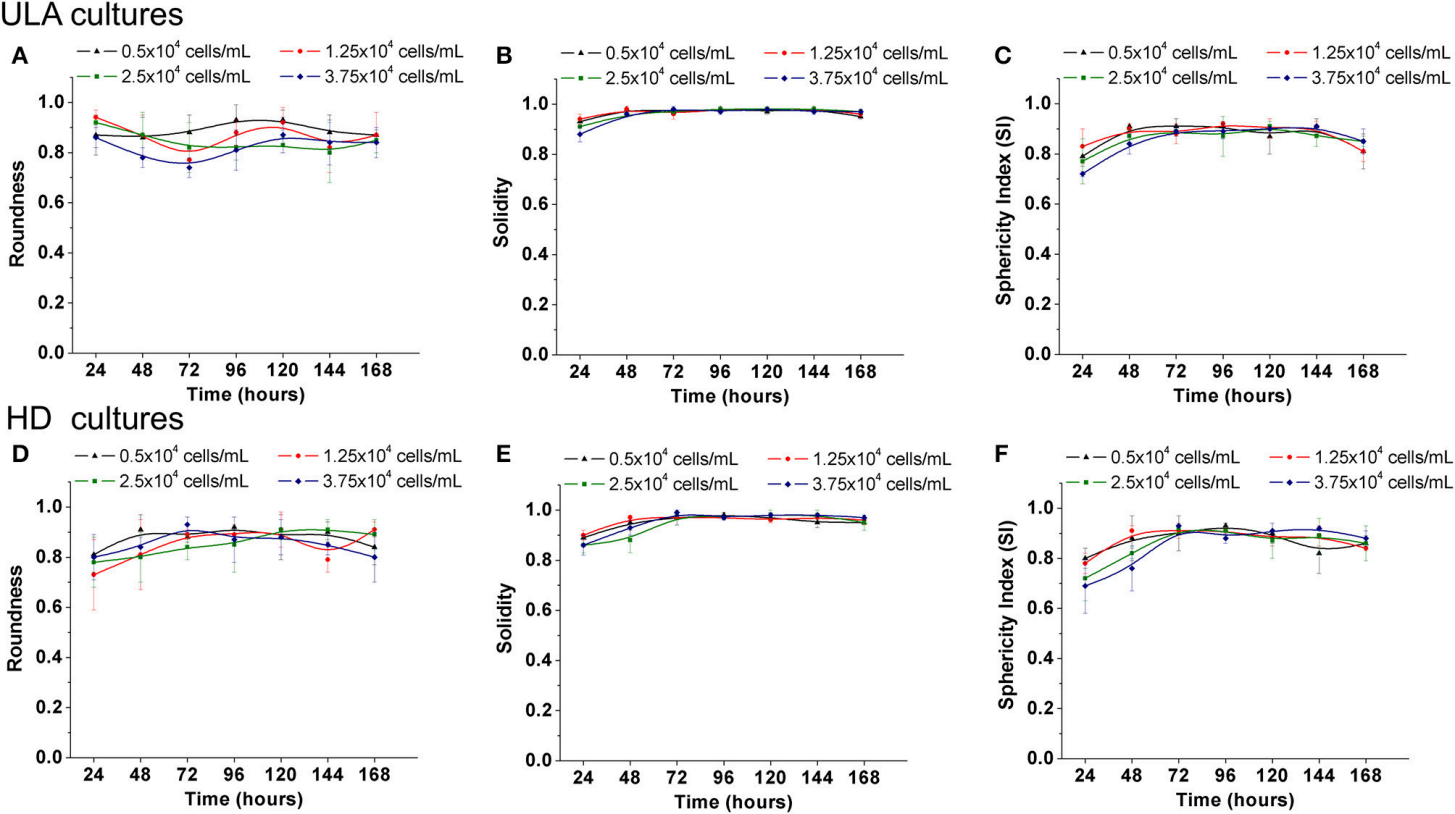
Shape parameters of RT4 spheroids throughout culture period in ULA **(A**–**C)** and HD **(D**–**F)** plates obtained using ImageJ software. **(A,D)** Roundness; **(B**,**E)**: Solidity; **(C**,**F)**: Sphericity Index (SI). Curves represent quadruplicate biological repeats and are displayed as mean ± SEM (*n* = 4).

The roundness of a spheroid is an indicator of the circularity of the projected area of the spheroids. It ranges from 0 to 1.0, and values closer to 1.0 indicate high circularly of the projected area. The roundness of the spheroids from the ULA plates oscillated between 0.75 and 0.95 during the whole experiment (Figure 3A). The best results were obtained for cultures seeded with 0.5 × 10^4^ cells/mL that indicate spheroids with roundness values over 0.90. In HD cultures, spheroids had roundness values over 0.85 and close to 0.90 for most of the time, independent of the cell-seeding concentration (Figure 3D). The low values of roundness observed during the first 24 h of the culture might be due to the lack of total compactness during this period. Throughout the culture, ECM secretions ensure total compactness or “spheroidization,” thus, increasing the roundness of the spheroids. A slight drop in spheroid roundness may occur over time, and is possibly associated with apoptosis and increase in the total spheroidal area (Gong et al., 2015). In general, both methods generated spheroids with a satisfactory circular shape when compared to results reported in literature (Raghavan et al., 2015).

The solidity is an indicator of the regularity of spheroids' surface. According to Santo et al. (2016), tumor spheroids are considered regular if solidity values are higher than 0.90 (no surface edges). The spheroids obtained from the ULA and HD cultures (Figures 3B,E, respectively) can be considered as regular, with solidity values higher than 0.95 after 48–72 h of culture regardless of the cell-seeding concentration used.

According to the sphericity index values, tumor aggregates could be also classified as spherical (SI ≥ 0.90) or non-spherical shape (SI < 0.90), the latter being subdivided into ellipsoidal, “8”-shaped, and irregular (Zanoni et al., 2016). At all cell seeding concentrations tested in the ULA method, the values of the sphericity index (SI) of the spheroids oscillated between 0.80 and 0.90 (Figure 3C). In the case of the HD method, spheroids from cultures seeded with 0.5 and 1.25 × 10^4^ cells/mL had Sphericity Index (SI) values ranging from 0.82 to 0.93 between 48 and 144 h (Figure 3F). During the same period, cultures seeded with 2.5 and 3.75 × 10^4^ cells/mL had SI values ranging from 0.76 to 0.93. In the latter situation, SI values higher than 0.80 were obtained only after 72 h of culture showing that these spheroids take longer to compact compared to spheroids with lower cell-seeding concentrations. Overall, spheroids from ULA and HD plates had values of SI close to 0.90, varying between ellipsoidal and spherical shape. Spheroids with an irregular shape may develop some aberrations such as the presence of two necrotic cores (Zanoni et al., 2016), which is rarely observed in spheroids with a spherical shape. Regular and well-rounded spheroids are more stable when employed in *in vitro* assays and exhibit less variability (Friedrich et al., 2009; Thoma et al., 2014; Weiswald et al., 2015).

### RT4 cells proliferate throughout 3D culture period, increasing the spheroid diameter

**Figure 4:**
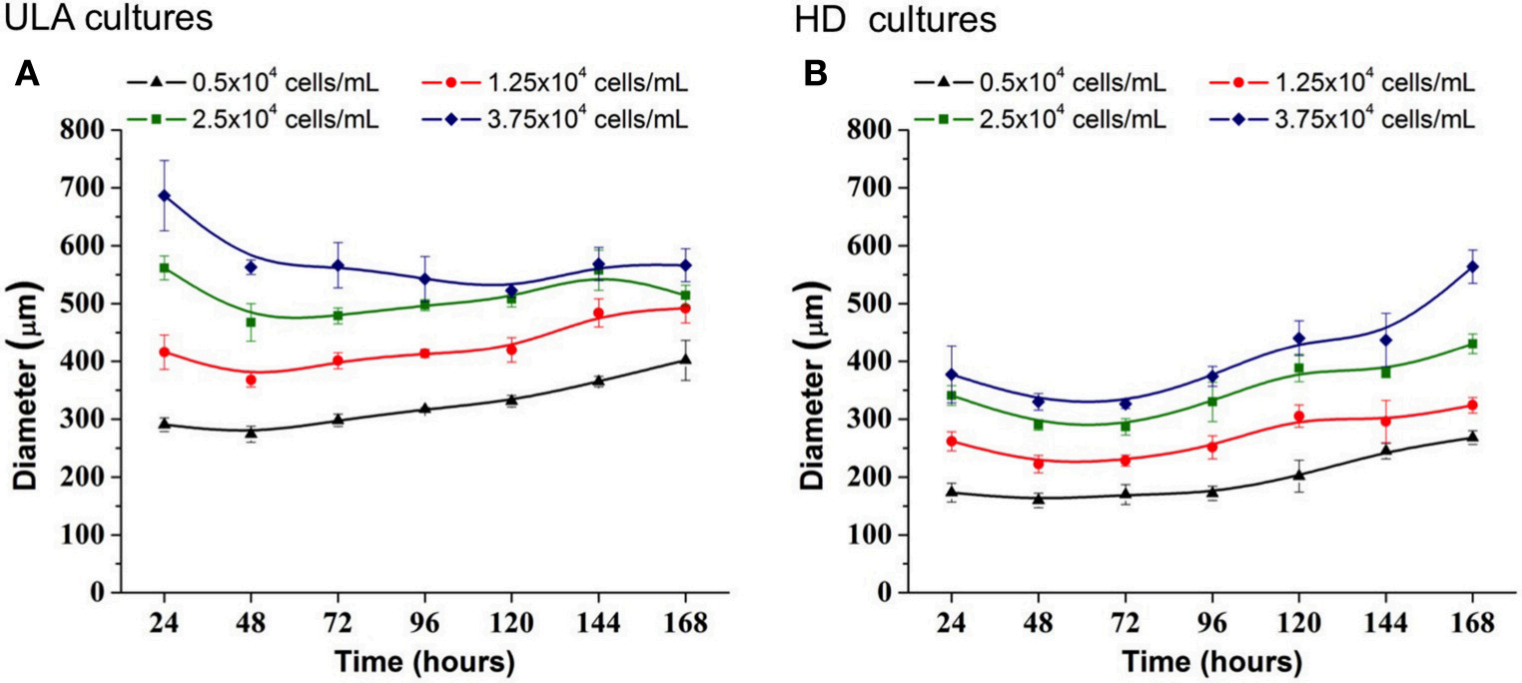
Increase in diameter of RT4 spheroids throughout culture period in ULA **(A)** and HD **(B)** plates obtained using ImageJ software. Curves represent quadruplicate biological repeats and are displayed as mean ± SEM (*n* = 4).

In the ULA method, the spheroid-diameter variation was high in cultures initiated with lower cell concentrations, 0.5 and 1.25 × 10^4^ cells/mL, ranging from 274.08 (±13.98) to 402.01(±34.01) μm and 368.17 (±12.40) to 492.14 (±25.32) μm, respectively (Figure 4A). On the other hand, a low variation in spheroid diameter was observed in cultures initiated with the two highest cell-seeding concentrations. At a seeding concentration of 2.5 × 10^4^ cells/mL, spheroids were obtained, whose diameter was higher than 450 μm at the beginning of the culture and reached values over 500 μm in 120 h. At seeding concentration of 3.75 × 10^4^ cells/mL, the spheroid diameter was larger than 500 μm, during the whole experiment.

3D cell cultures in HD plates were performed using a lower working volume when compared to ULA plates (40 μL for HD and 200 μL for ULA), as recommended by the manufacturer. The cell concentrations tested in HD culture were the same as those used in the ULA culture. However, the total number of cells seeded per well was lower in HD. For this reason, we obtained smaller spheroids by this method when compared to the ULA method (Figure 4B). Spheroids from cultures initiated with 0.5 × 10^4^ cells/mL reached a diameter of only 268.13 ± 11.80 μm by the end of the experiment. Cultures seeded with 1.25 × 10^4^ cells/mL generated spheroids with a diameter higher than 300 μm only after 120 h of culture. Cultures seeded with 2.5 and 3.75 × 10^4^ cells/mL generated spheroids with a diameter ranging from 340.92 ± 16.98 to 430.57 ± 17.12 μm and 330.18 ± 14.62 to 563.97 ± 28.53 μm, respectively. Spheroids with a diameter over 500 μm were not observed. The highest variation in spheroid diameters over 7 days was observed in cultures initiated with 2.5 and 3.75 × 10^4^ cells/mL.

The ideal spheroid diameter for use in cytotoxicity assays is widely discussed in literature, and no agreement has been reached yet. It is known that spheroids with a diameter of up to 200 μm are able to mimic 3D cell-cell and cell-matrix interaction, and they are frequently employed in drug testing (Fehlauer et al., 2005; Lambert et al., 2006; Friedrich et al., 2009). However, small spheroids are inappropriate for creating a pathophysiological condition, since they do not develop chemical or proliferative gradients (Larue et al., 2004; Hardelauf et al., 2011; Patra et al., 2013). Larger spheroids may contain a hypoxic core, and be more heterogeneous, with cells in different proliferation stages (Nath and Devi, 2016). They may also contain a necrotic core, which is be desirable for some drug testing approaches but could be problematic in others (Friedrich et al., 2009). Large tumors *in vivo* require the development of capillaries that allow blood perfusion in order to maintain cell functions at the center since passive diffusion of nutrients and O_2_ is insufficient (Alemany-Ribes and Semino, 2014). Without a perfusion system *in vitro*, spheroids with a diameter >500 μm develop a secondary necrosis core, which makes pathophysiological conditions complicated and difficult to control, subsequently, affecting the accuracy of the cytotoxicity assay (Inamdar and Borenstein, 2011; Wan et al., 2016). For these reasons, several researchers choose to work with spheroids of diameter ranging between 300 and 500 μm (Friedrich et al., 2009; Hardelauf et al., 2011; Wen et al., 2013; Ivanov et al., 2014; Patra et al., 2016; Wan et al., 2016). In the ULA method, 300–500 μm RT4 spheroids were obtained from cultures initiated with 0.5 and 1.25 × 10^4^ cells/mL. On the other hand, in the HD method, the cultures initiated with 2.5 and 3.75 × 10^4^ cells/mL generated spheroids with the appropriate diameter (300–500 μm).

**Figure 5:**
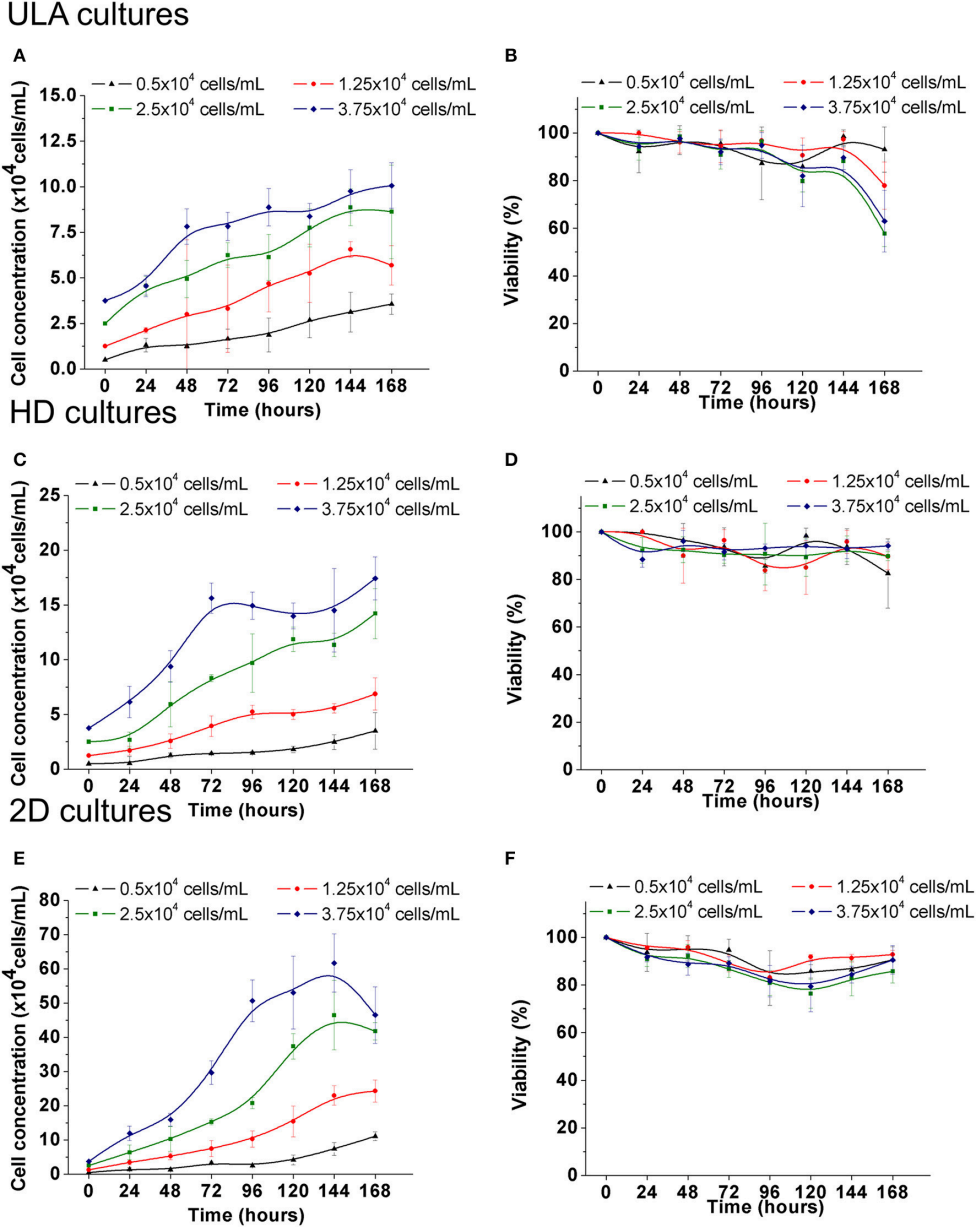
Cell growth and viability of RT4 cells throughout the culture period in ULA **(A**,**B)**, HD **(C**,**D)**, and monolayer cultures **(E**,**F)**. **(A**,**C,E)**: Cell concentration. **(B**,**D**,**F)**: Cell viability. Curves represent quadruplicate biological repeats and are displayed as mean ± SEM (*n* = 4).

Cell growth was observed at all cell-seeding concentrations for both the ULA and HD methods. In ULA cultures a fold increase (FI) of 7.0 ± 1.11, 4.5 ± 0.87, 3.5 ± 1.0, and 2.7 ± 0.33 in cell concentration was observed for cultures initiated with 0.5, 1.25, 2.5 and 3.75 × 10^4^ cells/mL, respectively (Figure 5A). Viability of over 90% was maintained during most of the culture period in cultures seeded with 0.5 and 1.25 × 10^4^ cells/mL (Figure 5B). The viability of spheroids from the two highest cell-seeding concentrations, 2.5 and 3.75 × 10^4^ cells/mL was maintained near 90% only during the first 96 h, and decreased to values lower than 70% by the end of the culture period.

Spheroids cultured in HD plates showed a FI of 7.0 ± 3.34, 5.5 ± 1.82, 5.65 ± 0.91, and 4.64 ± 0.52 in cell concentration in cultures seeded with 0.5, 1.25, 2.5, and 3.75 × 10^4^ cells/mL, respectively (Figure 5C). No significant differences were observed between FI numbers of ULA and HD cultures. Viability of over 90% was maintained during the whole experiment in cultures seeded with 2.5 and 3.75 × 10^4^ cells/mL (Figure 5D). Cultures seeded with 0.5 and 1.25 × 10^4^ cells/mL showed a small drop in viability after 72 h of culture, but all cultures showed viability of over 80% by the end.

When cultured in monolayers (2D), RT4 cells showed a typical *in vitro* cell-growth profile, having the lag, exponential, and stationary phases in cultures seeded with 2.5 and 3.75 × 10^4^ cells/mL (Figure 5E). This cell-growth profile was not observed in 3D cultures. 2D cultures showed a FI of 22.1 ± 2.61, 19.5 ± 2.58, 16.7 ± 1.0, and 12.4 ± 2.2 in cell concentration when seeded with 0.5, 1.25, 2.5, and 3.75 × 10^4^ cells/mL, respectively (Figure 5F). These values were higher than those observed for 3D cultures (One-way ANOVA with *post-hoc* test Tukey HSD, *P* < 0.001). Under all conditions, viability was maintained close to or higher than 90% during the first 72 h, and showed a small drop after this point, but values over 80% were maintained by the end (Figure 5B).

The differences in growth rate observed between 3D and 2D cultures with RT4 cells are also observed in other cell lines (Maria et al., 2011; Chitcholtan et al., 2012; Luca et al., 2013). All cells in 2D cultures receive equal amount of nutrients and growth factors from the medium, since they attach themselves to a flat surface, forming a monolayer (Huang et al., 2013). During the exchange of culture medium, most of necrotic cells are removed, and therefore, proliferating cells are predominant in 2D cultures (Edmondson et al., 2014). On the other hand, 3D spheroids consist of cells in different stages with viable and proliferating cells only in the outer layers. This feature results in cell growth dynamics with initial cell proliferation followed by delayed growth, which is commonly observed in *in vivo* tumors (Sutherland, 1988). Therefore, when cultured as spheroids, RT4 cells were able to exhibit the growth characteristics observed in bladder tumor cells *in vivo*.

**Figure 6:**
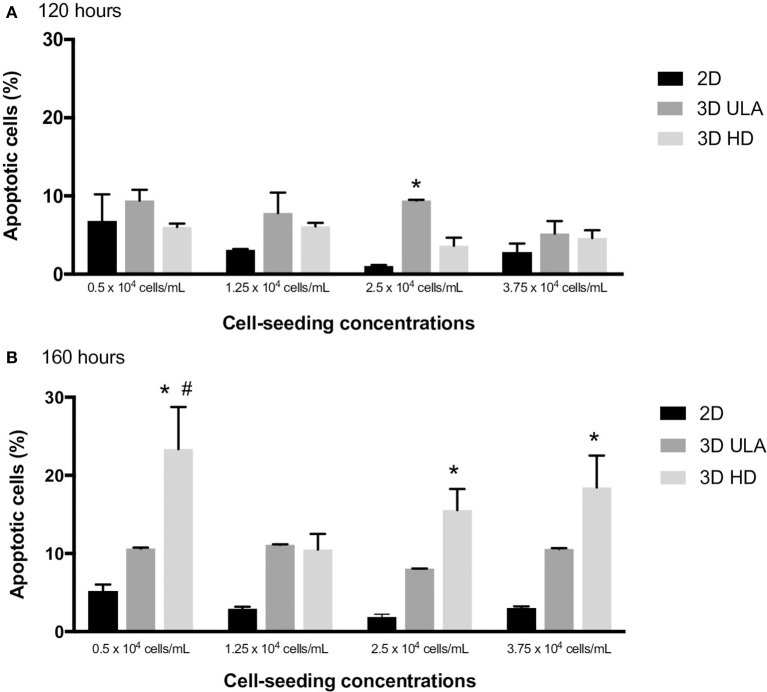
Number of apoptotic cells (%) in 2D and 3D cultures at 120 **(A)** and 168 h **(B)**. Curves represent triplicate biological repeats and are displayed as mean ± SEM (*n* = 3). One-way ANOVA statistic method with *post-hoc* test Tukey HSD (^*^P < 0.05 vs. 2D; ^#^*P* < 0.05 vs. 3D ULA).

Apoptotic cells in 2D and in 3D cultures were also evaluated by flow cytometry using Annexin V-FITC as the apoptosis marker. The percentage of apoptotic cells at 120 and 168 h of culture in each situation is shown in Figure 6. For 120-h cultures, statistical difference was only observed between 2D and 3D ULA cultures seeded with 2.5 × 10^4^ cells/mL (One-way ANOVA with *post-hoc* test Tukey HSD, *P* < 0.05). In case of 168-h cultures, we observed a significant higher number of apoptotic cells in HD cultures seeded with 0.5, 2.5, and 3.75 × 10^4^ cells/mL when compared with 2D cultures (One-way ANOVA with *post-hoc* test Tukey HSD, *P* < 0.05). Apoptosis may occurs over time in these systems due to the lack of a vasculature (Hirschhaeuser et al., 2010; Gong et al., 2015; Stock et al., 2016). A high number of apoptotic cells could be also an indicator of dysfunctional mechanisms of cell-cell and cell-ECM interactions, since these interactions operate to control apoptosis (Zahir and Weaver, 2004).

### As with growth, the metabolic activity of RT4 cells is low in 3D cultures

**Figure 7:**
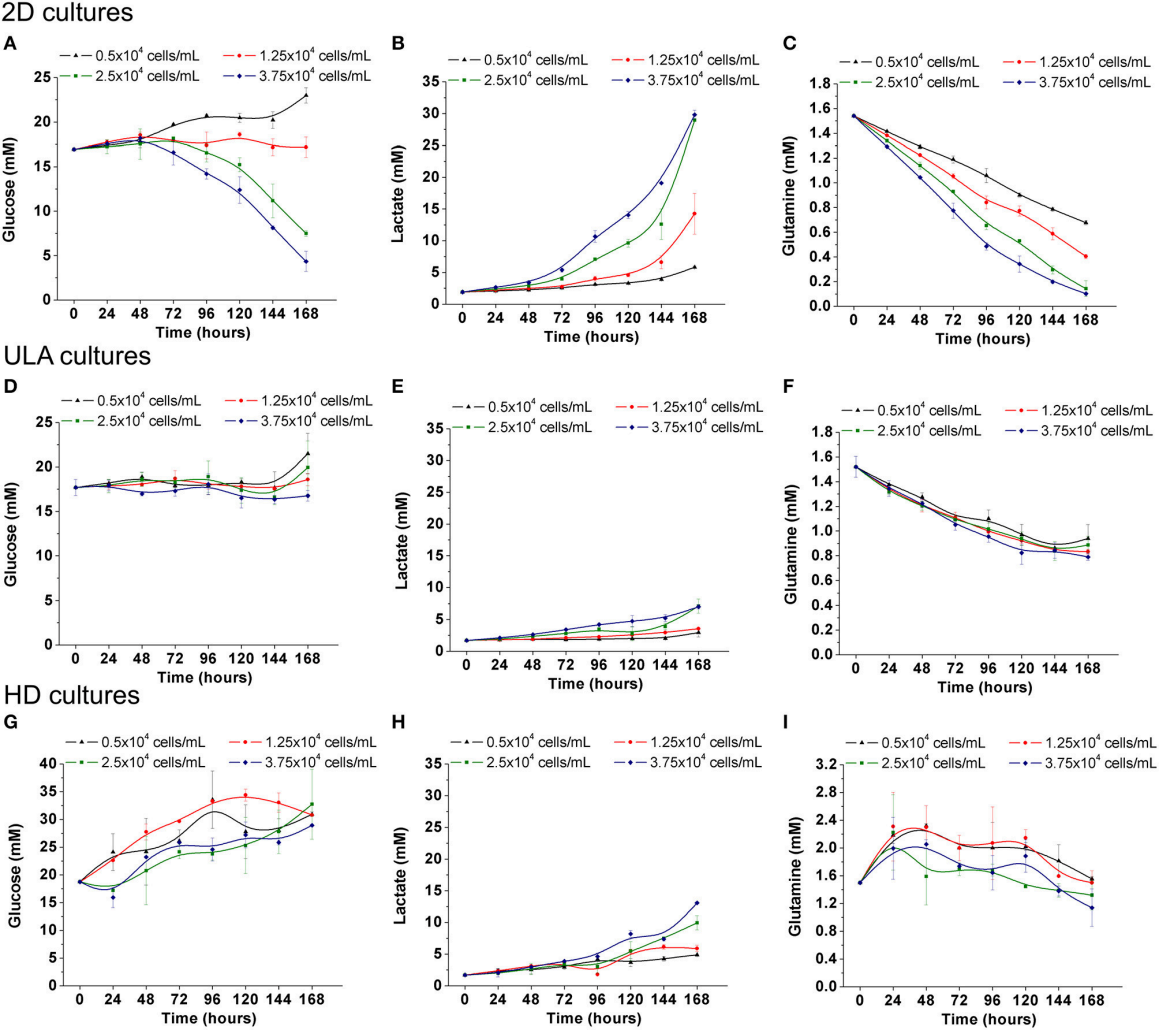
Metabolism of RT4 cells in 2D culture **(A–C)** and ULA **(D,F,G)** and HD **(G–I)** cultures. **(A**,**D**,**G)** Glucose concentration; **(B**,**E**,**H)** Lactate concentration; **(C,F,I)** Glutamine concentration. Curves represent triplicate biological repeats and are displayed as mean ± SEM (*n* = 3).

The metabolic activity of RT4 cells in 3D cultures was also characterized in this study, and compared with the results obtained under 2D culture conditions. Under 2D, the consumption of glucose by RT4 cells was only observed in cultures with the two highest cell-seeding concentrations (Figure 7A). In cultures seeded with 2.5 and 3.75 × 10^4^ cells/mL, the concentration of glucose decrease from 17 ± 0.16 mM to 7.5 ± 0.32 and 4.35 ± 1.12 mM, respectively. As lactate is a byproduct of glucose metabolism, the concentration of lactate reached the highest level, about 30 mM, in the cultures with the higher glucose consumption (2.5 and 37.5 × 10^4^ cells/mL; Figure 7B). On the other hand, a decrease in glutamine concentrations in the medium was observed, regardless of the cell seeding concentration (Figure 7C). Glutamine levels decreased from 1.54 ± 0.01 mM to 0.68 ± 0.02, 0.40 ± 0.02, 0.14 ± 0.06, and 0.10 ± 0.01 mM in cultures initiated with 5, 1.25, 2.5, and 3.75 × 10^4^ cells/mL, respectively. Lea and collaborators have already described that RT4 cells exhibited glutamine-dependent cell growth, unlike other bladder cancer cell lines, wherein glucose is the main energy source (Lea et al., 2015).

The consumption of nutrients of RT4 cells in 3D cultures was lower when compared to 2D culture. This situation is expected once metabolism can be altered depending on the type of cancer cells, invasiveness and culture conditions (Conde et al., 2015). Glucose was not consumed neither in ULA cultures nor HD cultures (Figures 7D,G) resulting in a small production of lactate in the culture medium (Figures 7E,H). The variations in glucose levels in the 3D cultures, mainly in HD method, can be explained by the considerable medium evaporation (around 50% after 7 days of culture). Although the consumption of glutamine in 3D cultures was lower than 2D cultures, a preference for glutamine instead of glucose was also observed (Figures 7F,I). The reduction in metabolic activity of cells within multicellular spheroids was also observed in other studies (Desoize and Jardillier, 2000). This must be related to the fact that metabolic enzymes involved in glycolysis and glucose transporters are downregulated when cells are organized into 3D spheroids (Werner and Werb, 2002). Another interesting fact is that the 3D architecture may induce a sustained effect on cellular metabolism, since some works have shown that single cells derived from dissociated spheroids exhibited consistently reduced metabolism (Zahir and Weaver, 2004). The decreased dependence on glycolysis or enhanced oxidative phosphorylation would provide cells less sensitive to eventual changes in nutrition when cultured in 3D spheroids (Zahir and Weaver, 2004).

### RT4 cells of 3D cultures showed higher drug resistance compared to 2D cultures

**Figure 8:**
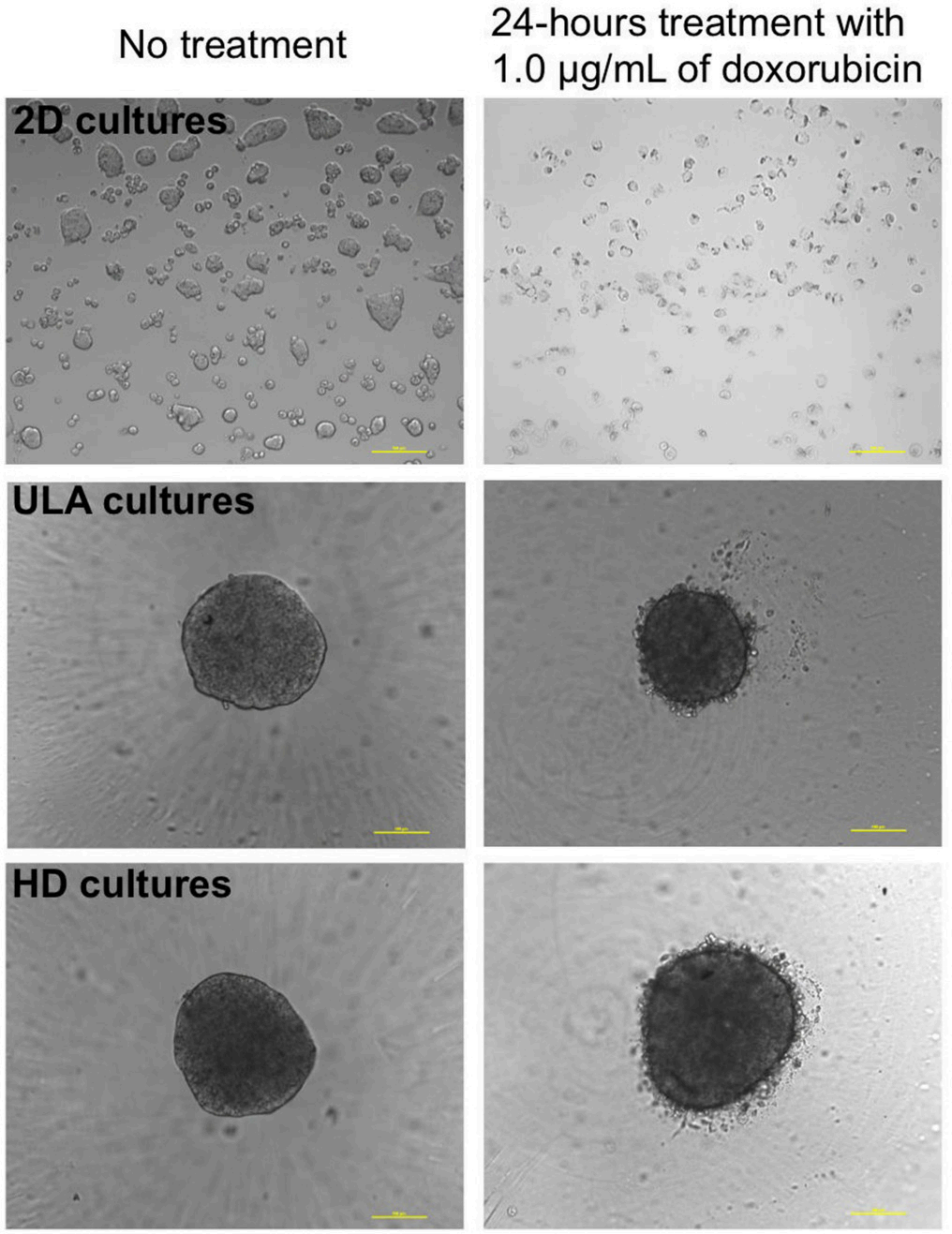
Phase contrast microscopic images of 2D and 3D (ULA and HD) cultures of RT4 cells before and after treatment with 1.0 μg/mL of doxorubicin (10X objective, scale bar = 100 μm).

The drug response study was conducted with spheroids from 72 h-cultures with cell-seeding concentrations of 0.5 and 1.25 × 10^4^ cells/mL for ULA plates, and 2.5 and 3.75 × 10^4^ cells/mL for HD plates. Under these conditions, spheroids with the desired diameter (300–500 μm) and shape parameters were obtained. Spheroids and monolayer cultures were treated with different concentrations of doxorubicin: 4.0, 2.0, 1.0, 0.5, 0.25, and 0.125 μg/mL. Doxorubicin is a chemotherapeutic agent used in the treatment of several types of tumors (Carter and Comis, 1975), including bladder cancer. Therefore, doxorubicin was chosen to evaluate the sensitivity of RT4 cells in the 3D cultures. Images obtained by phase contrast microscopy, after the 24-h treatment with doxorubicin showed a cloud of debris and dying cells surrounding the spheroids at all drug concentrations, while most of cells in 2D cultures were dead, as represented in Figure 8.

**Figure 9:**
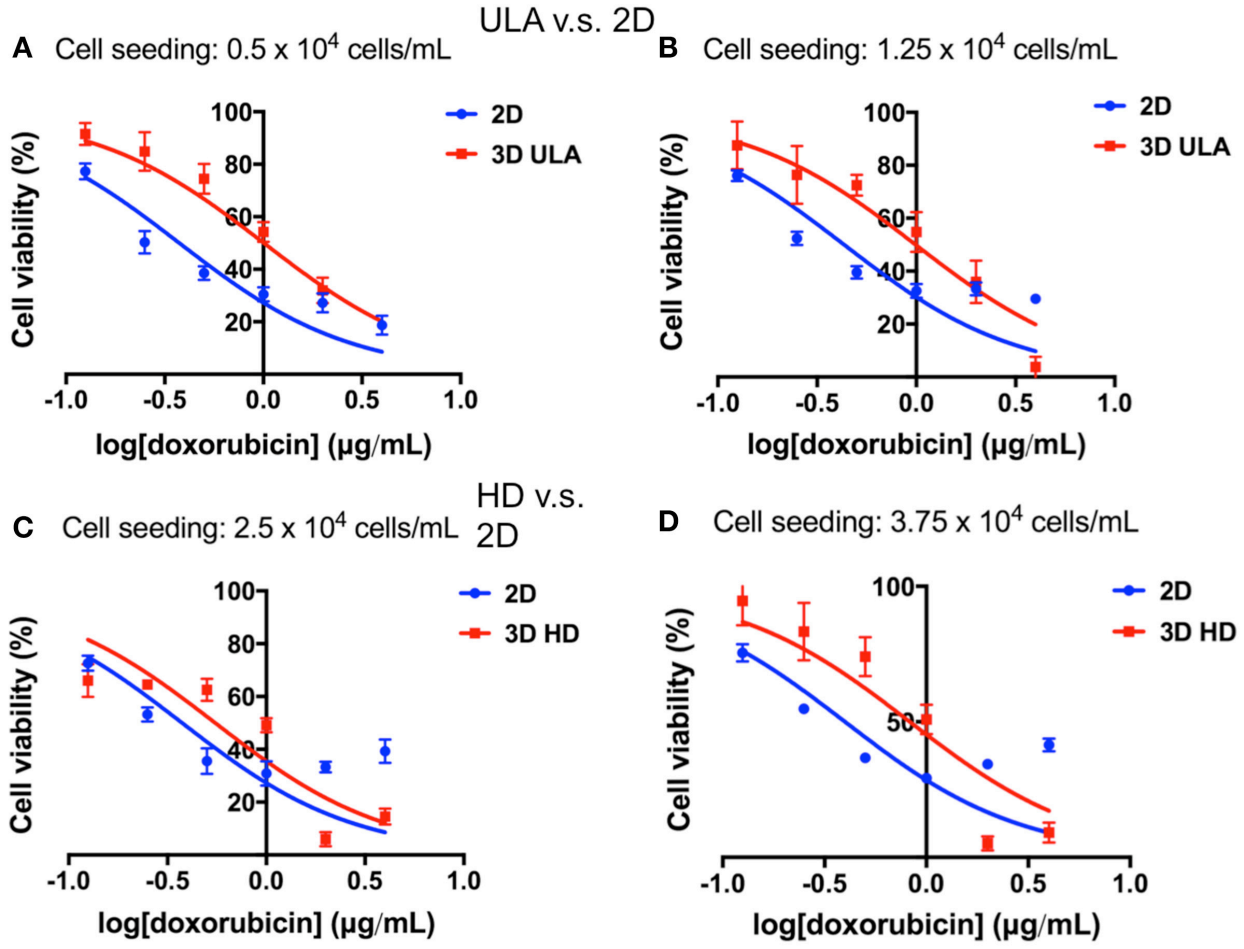
Concentration-response curves: cell viability (%) plotted against the logarithm of the doxorubicin concentration (μg/mL). **(A)** 2D and 3D ULA cultures seeded with 0.5 × 10^4^ cells/mL; **(B)** 2D and 3D ULA cultures seeded with 1.25 × 10^4^ cells/mL; **(C)** 2D and 3D HD cultures seeded with 2.5 × 10^4^ cells/mL; **(D)** 2D and 3D HD cultures seeded with 3.75 × 10^4^ cells/mL. Curves represent sextuplicate biological repeats and are displayed as mean ± SEM (*n* = 6).

Differences in drug response between cells of 2D and 3D cultures could be observed in all situations as shown in Figure 9. The inhibitory dose-response curve fit showed that the activity of doxorubicin was higher in 2D cultures than in 3D cultures. These results were confirmed by the IC_50_ values (Table 1). In both 3D conditions, ULA and HD, the IC_50_ values were statistically higher than those observed in 2D cultures (*P* < 0.001 for cultures seeded with 0.5, 1.25, and 3.75 × 10^4^ cells/mL, *P* < 0.05 for cultures seeded with 2.5 × 10^4^ cells/mL, extra-sum-of-squares *F-*test).

**Table 1:** IC_50_ (μg/mL) assayed for doxorubicin resistance in 2D and 3D cultures.

	**Cell-seeding concentrations (×10^4^ cells/mL)**							
	**0.5**		**1.25**		**2.5**		**3.75**	
**Culture model**	**2D**	**3D ULA**	**2D**	**3D ULA**	**2D**	**3D HD**	**2D**	**3D HD**
IC_50_ (μg/mL)	0.37	1.0^**^^##^	0.43	0.99^**^^##^	0.37	0.55^*^	0.4	0.83^**^^#^
Confidence Interval 95%^a^	0.32–0.44	0.84–1.2	0.34–0.54	0.83–1.18	0.28–0.49	0.44–0.68	0.30–0.52	0.66–1.04

Extra-sum-of-squares statistic method with F test:

* P < 0.05 vs. 2D cultures with the same cell seeding;

** P < 0.001 vs. 2D cultures with the same cell seeding;

# P < 0.05 vs. 3D HD with cell seeding of 2.5 × 10^4^cells/mL;

## P < 0.001 vs. 3D HD with cell seeding of 2.5 × 10^4^cells/mL;

a *Confidence interval (95%) related to the comparative analysis between 3D and 2D cultures with the same cell seeding*.

Some studies have compared the effect of anticancer drugs in 2D and 3D cultures using different cancer cell lines (Adcock et al., 2015; Baek et al., 2016; Oliveira et al., 2016; Wan et al., 2016). Most of them have already pointed that tumor cells in 2D cultures often die after treatment with the anticancer drug, while cells in 3D cultures are often more resistant (Ponce de León and Barrera-Rodríguez, 2005; Hongisto et al., 2013; Breslin and O'Driscoll, 2016). These differences can be attributed to several reasons. The first reason is concerned with the difference in the accessibility of the drug to cells (Edmondson et al., 2014) once exposure to medium/drugs is different between 2D and 3D cultures. Cells in monolayer are uniformly exposed to drugs present in the medium, whereas drug may not be able to fully penetrate the 3D structure (Kim, 2005; Yip and Cho, 2013). Besides diffusion, the pH of the cells is also important since low pH reduces drug uptake, thus, contributing to drug resistance (Swietach et al., 2012). It has been shown that the hypoxic microenvironment at the core of the spheroids leads to the activation of genes involved in cell survival and drug resistance (Trédan et al., 2007). Since doxorubicin promotes cellular death through the formation of reactive oxygen species (Wartenberg et al., 2005), this drug would have a lower therapeutic efficacy under hypoxic conditions. The differences in cell phases are also important in drug resistance. In monolayer cultures, there is a predominance of proliferating cells, whereas in 3D cultures, there is a combination of cells at different stages (heterogeneity). This fact is important since some drugs such as doxorubicin are effective only against actively proliferating cells (Chitcholtan et al., 2012; Wen et al., 2013). Finally, the fifth reason is the difference in expression levels of oncogenes that may be involved in drug action and affect its efficacy (Edmondson et al., 2014).

### Comparative analysis of the ULA and HD methods

A comparative analysis of the main results obtained employing ULA and HD plates for 3D culture of RT4 cells is presented in Table 2. Both methods, ULA and HD, generated RT4 spheroids between 24 and 48 h of culture (spheroidization time) with a round-type morphology and the presence of compact surface containing microvilli. Regarding the shape, we generated ellipsoidal/spherical spheroids (sphericity index around 0.90) with a regular surface (solidity higher than 0.95) in both methods. On the other hand, spheroids from ULA plates showed roundness values more close to 1.0 than spheroids from HD plates.

**Table 2:** Comparison of the results obtained for 3D spheroids of RT4 cells generated by forced floating and hanging drop methods.

	**3D methods**	
	**ULA**	**HD**
Spheroidization time	Between 24 and 48 h	Between 24 and 48 h
Morphology	Round-type	Round-type
Presence of microvilli	Yes	Yes
Roundness	0.75–0.95	0.85–0.90
Solidity	Regular surface (>0.95)	Regular surface (>0.95)
Sphericity Index	Ellipsoidal/Spherical shape	Ellipsoidal/Spherical shape
Diameter ranging: 300–500 μm	Cultures seeded with 0.5 and 1.25 × 10^4^ cells/mL	Cultures seeded with 2.5 and 3.75 × 10^4^ cells/mL
Fold increase (FI)	Lower than 2D^a^ Similar to HD	Lower than 2D^a^ Similar to ULA
Number of apoptotic cells	120 h: similar to 2D	120 h: similar to 2D
	168 h: similar to 2D	168 h: higher than 2D^b^
Metabolism	Reduced compared to 2D	Reduced compared to 2D
IC_50_ value	Higher than IC_50_ of 2D^c^	Higher than IC_50_ of 2D^d^
Cell plating	Easy	Difficult
Culture maintenance	Easy	Difficult
Real-time monitoring	Easy	Difficult
Medium change	Trivial	Not trivial
Possibility of performing assays directly in the plates	Yes	No

a *One-way ANOVA statistic method with post-hoc test Tukey HSD **(**P < 0.001)*.

b *One-way ANOVA statistic method with post-hoc test Tukey HSD (P < 0.005)*.

c *Extra-sum-of-squares statistic method with F-test (P < 0.001)*.

d *Extra-sum-of-squares statistic method with F-test (P < 0.05 for HD cultures seeded with 2.5 × 10^4^ cells/mL and P < 0.001 for HD cultures seeded with 3.75 × 10^4^ cells/mL)*.

The growth of cells in the spheroids was assessed according to changes in diameter, cell number, and metabolism. This information was important in selecting the cell-seeding concentration that was used for the drug resistance assay. A reduced cell growth was observed in spheroids compared to 2D cultures. HD plates showed a significant higher number of apoptotic cells when compared to 2D in 168-h cultures. Neither nutrient exhaustion nor accumulation of lactate to inhibitory levels was observed in the culture medium. The IC50 values obtained in both 3D cultures were similar and significantly higher than those obtained to 2D cultures.

The necessity to transfer the spheroids from the HD plates to the conventional 96-well plates to carry out the cytotoxicity assay was considered a limitation of this method. Maintaining the culture conditions of the HD method was also considered a limitation since the low working volume resulted in the quick evaporation of culture medium, and any mechanical shock during manipulation and culture would shatter the spheroids. The ULA method, on the other hand, offers a simpler workflow, and can be easily used in high-through put screening platforms. Based on these limitations, we considered the forced floating method using ULA plates as the most suitable and straightforward method to generate RT4 spheroids for cytotoxicity assays. Our findings corroborate with the literature reporting promising results using ULA method with different types of tumor cells (Vinci et al., 2012; Howes et al., 2014; Feng et al., 2017; Selby et al., 2017).

## Conclusion

RT4 cells were able to be cultured under 3D conditions in both ULA and HD plates. RT4 spheroids exhibited reduced cell growth and metabolism as well as higher drug-resistance when compared to 2D cultures. Therefore, we confirmed that these 3D *in vitro* models were more representative of the *in vivo* tumor microenvironment. However, the forced floating method using ULA plates was demonstrated to be more robust and reliable than the HD method. Therefore, we considered the forced floating method using ULA 96-well round-bottomed plate as the most suitable and straightforward method to generate RT4 spheroids for cytotoxicity assays. The results presented here can contribute to the improvement in the standardization of 3D spheroids cultures ensuring the application of these models on mainstream drug screening process.

## Author contributions

RA performed all the experiments. MM and PM helped in the drug sensitivity assay and contribute to manuscript writing. KS guided the work and was a major contributor in writing the manuscript. All authors read and approved the final manuscript.

### Conflict of interest statement

The authors declare that the research was conducted in the absence of any commercial or financial relationships that could be construed as a potential conflict of interest.

## Abbreviations

ECM: Extracellular Matrix
FI: Fold Increase
HD: Hanging Drop
SI: Sphericity Index
ULA: Ultra-low Attachment.
